# Vitamin D inhibits apoptosis in THP-1 cells infected with mycobacterium tuberculosis through TNF signaling pathway

**DOI:** 10.3389/fimmu.2025.1525922

**Published:** 2025-05-06

**Authors:** Yusheng Yang, Jiezhong Deng, Pan Liu, Jinyue He, Jiulin Tan, Bo Yu, Yun Bai, Fei Luo, Jianzhong Xu, Zehua Zhang

**Affiliations:** ^1^ Department of Orthopedic, Southwest Hospital, Army Medical University, Chongqing, China; ^2^ Institute of Orthopedic Trauma of Chinese People’s Liberation Army (PLA), The 80th Group Army Hospital, Weifang, Shandong, China

**Keywords:** *Mycobacterium tuberculosis*, vitamin D, TNF, apoptosis, inflammation

## Abstract

Vitamin D (VD) has been extensively associated with the resistance against tuberculosis (TB); however, the mechanism underlying the reduction in TB susceptibility by VD remains uncertain. In our prior investigation, we discovered the relationship between VD and *mycobacterium tuberculosis M.tb*-induced aberrant osteoclastogenesis. Here we report that VD diminishes apoptosis in *M.tb*-infected THP-1 cells through tumor necrosis factor (TNF) signaling pathway. This novel perspective contributes to the elucidation of the intricate relationship between VD and tuberculosis. In this study, THP-1 cells were infected with the *Mycobacterium tuberculosis H37Rv* strain (*M.tb*) for 4h at a MOI of 1 and then treated with 1,25-dihydroxy vitamin D (1,25(OH)_2_D_3_) (10^-6^, 10^-8^, 10^-10^M) for 1d respectively. RNA sequencing (RNA-seq) was performed, and differential expression analysis was conducted by the R package edgeR. Immunofluorescence (IF) and immunohistochemistry (IHC) techniques were employed for VDR, TNFR1 and TUNEL in TB patients and serum levels of TNF-α and IL6 were measured simultaneously. Furthermore, the utilization of western blot and qRT-PCR techniques was employed to investigate the impact of VD on pivotal molecules involved in the TNF signaling pathway. In addition, Bacillus Calmette-Guérin (BCG, ATCC 35734, derived from *M.bovis*) and VD were administrated by tail vein and articular cavity injection *in vivo*. Our findings revealed a robust responsiveness of the TNF signaling pathway to *M.tb*-induced inflammation, resulting in elevated expression of TNF-α, IL-6, and severe apoptosis. VD exhibited significant inhibitory effect on *M.tb*-induced inflammation and apoptosis both *in vitro* and *in vivo*. This study offers novel insights for vitamin D in the study of tuberculous bone destruction.

## 1 Introduction

Tuberculosis (TB) is a highly contagious disease that significantly contributes to poor health worldwide, ranking among the top 10 causes of death. The emergence of the novel coronavirus disease (COVID-19) poses new challenges for TB control (1). *Mycobacterium tuberculosis* (*M.tb*) is the etiological agent of tuberculosis and is spread through droplet spread when patients cough. It is estimated that around one-quarter of the world’s population has been infected with *M.tb*, especially in developing countries (2). It predominantly affects the lungs, leading to pulmonary tuberculosis, but can also manifest as extrapulmonary tuberculosis, including lymphatic tuberculosis, pleural tuberculosis, and osteoarticular tuberculosis (3). Spinal tuberculosis, accounting for 50% of all cases, is the most prevalent form of osteoarticular tuberculosis (4, 5), which is commonly found in southwest China (Chongqing, Guizhou, Sichuan, etc.) with high incidence of tuberculosis (6, 7).

Macrophages play an essential role in the innate immune system both in vertebrates and invertebrates, serving as the primary defense against intracellular parasites before transitioning to the adaptive immune response (8). During pulmonary infection, *M.tb* initially encounters the host’s innate immunity, primarily alveolar macrophages (9). However, the phagocytosis of *M.tb* hinders the binding of phagocytic vesicles to lysosomes through immune evasion mechanisms (10, 11). Macrophages must maintain a delicate balance between resting homeostasis function, inflammatory function and cell death (12). In the case of infection within macrophage cells, insufficient cell death can result in the spread of *M.tb*, while excessive cell death will impede their abilities to function (13). Though inflammation within the granuloma is necessary to prevent *M.tb* dissemination, excessive inflammatory responses cause decreased disease tolerance (14, 15). Furthermore, different virulence of *M.tb* can either promote or inhibit macrophage apoptosis, which reveals that apoptosis is a host innate defense mechanism against TB (16). Tumor necrosis factor (TNF) primarily exerts its effects through tumor necrosis factor receptor (TNFR) and is involved in cell communication, cell differentiation and cell death (17). It’s also a component of the extrinsic pathway that triggers apoptosis (4). However, the role of TNF pathway in *M.tb*-induced apoptosis remains unclear.

Vitamin D, a lipid-soluble vitamin, exerts its effects on cells through vitamin D receptor (VDR) and can regulate the bone homeostasis (18, 19). Vitamin D can be metabolized to active 1,25-dihydroxy vitamin D (1,25(OH)_2_D_3_) *in vivo*. Researches indicated that vitamin D intake can decrease the susceptibility to tuberculosis (20), but the underlying mechanisms remain unclear. *M.tb* can use lipid droplets in macrophages as a carbon source for replication (21). Activation of VDR enhances the expression of fatty acid synthase and promotes lipogenesis, leading to decreased lipolysis and lipid deposition (22–24). In our previous study, we demonstrated that vitamin D inhibited the abnormal activation of osteoclasts induced by *M.tb* (25). Additionally, vitamin D serves as a crucial regulator of runt-associated transcription factor 2 (RUNX2) and promotes osteoblast maturation in mesenchymal stem cells (MSCs) (26). Hence, vitamin D effectively inhibits the growth of intracellular *M.tb* while also participates in restoring the damaged bone matrix.

The current study elucidates the mechanism that 1,25(OH)_2_D_3_ inhibits macrophage apoptosis during *M.tb* infection. Furthermore, 1,25(OH)_2_D_3_ can suppress the inflammatory response triggered by *M.tb* infection and reduce the inflammatory factors, including TNF-α, via the NF-κB signaling pathway, which might provide a new idea for the treatment of TB.

## 2 Results

### 2.1 The infection of *M.tb* activates the TNF pathway in THP-1 cells

**Figure 1:**
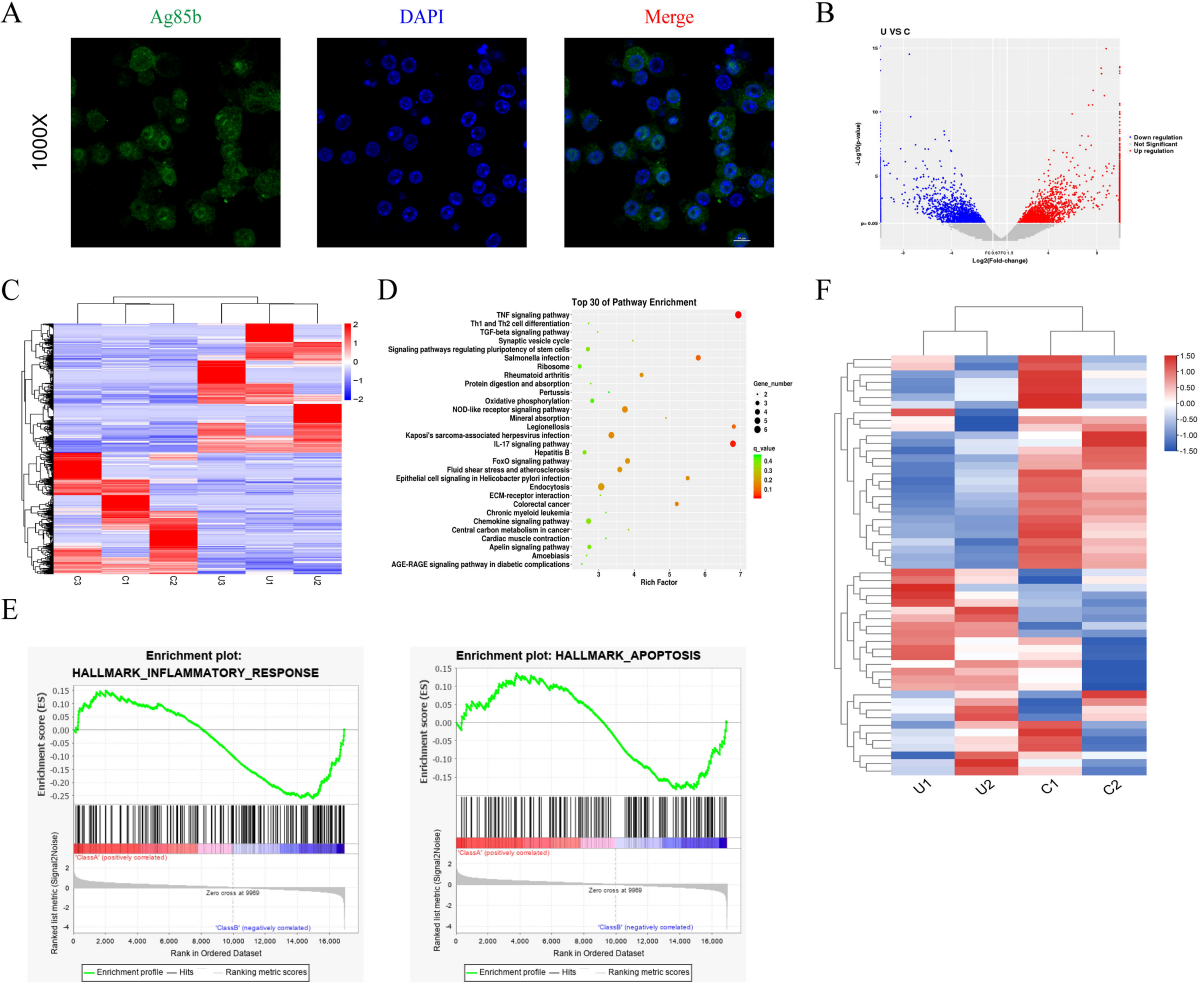
Immunofluorescence results and high-throughput sequencing results of THP-1 infected with *M.tb*. **(A)** Immunofluorescence staining for Ag85b of THP-1 cells infected by *M.tb*. Scale bars, 20 µm. **(B, C)** The volcano plot and heat map analysis showed the gene profiling expression in THP-1 infected with *M.tb*. **(D)** The KEGG pathway enrichment. **(E)** GSEA plots for selected hallmark pathways showing the changes in the pathway after *M.tb* infection. **(F)** The heat map shows the up and downregulated genes in the TNF pathway after *M.tb* infection.

To examine alterations in gene transcription during *M.tb* infection, we constructed an *in vitro* bacterial infection model (27). A specific antibody was employed to perform immunofluorescence staining of Ag85B, a conserved *M.tb*-specific protein (28). The findings revealed a distinct Ag85B positive signal in THP-1 cells (**Figure 1A**), indicating the ability of macrophages to phagocytose *M.tb*. High-throughput sequencing was conducted to identify the effector molecules regulated by *M.tb*. A total of 30971 genes were changed and visualized in a scatter plot (**Figure 1B**). The gene expression was visualized through heatmap analysis (a total of 1811 DE-genes were identified, including 934 upregulated and 877 downregulated genes) (**Figure 1C**). Kyoto Encyclopedia of Genes and Genomes (KEGG) analysis was performed and the top 30 were displayed (**Figure 1D**). Gene Set Enrichment Analysis (GSEA) was performed to identify differentially regulated gene sets, the inflammatory and apoptosis signaling pathway were enriched (**Figure 1E**). As the TNF signaling pathway is the top enriched pathway and plays a crucial regulatory role in macrophages, we reasonably hypothesized that the TNF signaling pathway plays a vital role in *M.tb*-infected macrophages and selected it for further verification. The genes related to the TNF signaling pathway were visualized using a heat map (**Figure 1F**). The results above indicate that the TNF signaling pathway exhibited the most significant difference during *M.tb* infection.

### 2.2 Analysis of inflammatory factors and apoptosis rate in osteoarticular tuberculosis patients

**Figure 2:**
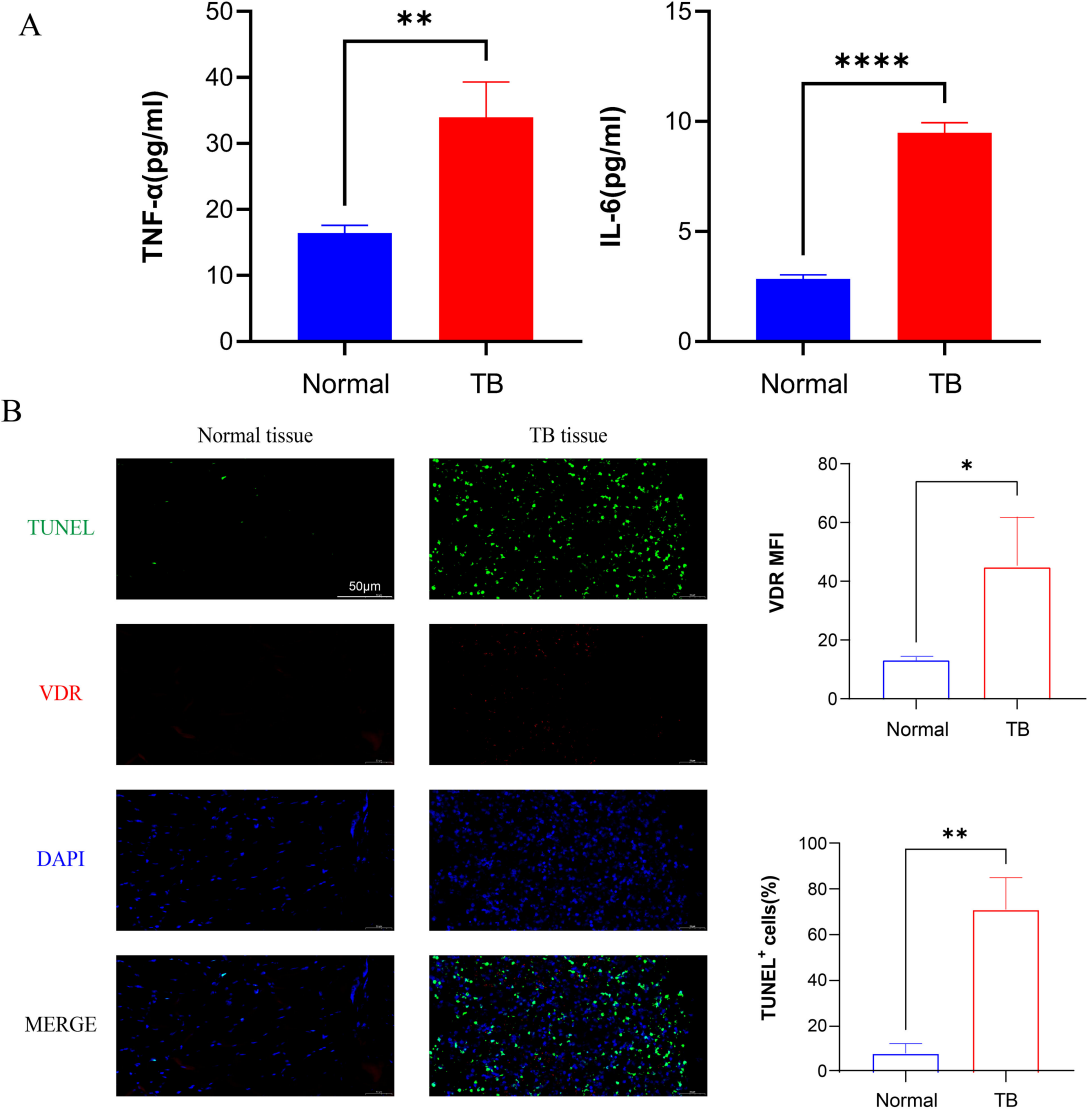
*M.tb* infection promotes the secretion of inflammatory factors and tissue apoptosis. **(A)** Serum concentration factors TNF-α and IL-6 levels of healthy volunteers and TB patients. (n=3) **(B)** Immunofluorescence staining of TUNEL (green), VDR (red) and DAPI (blue) from TB patients. Scale bars, 50µm. (n=3) Student’s t test was used to calculate p values. *p < 0.05, **p < 0.01, ****p<0.0001.

TNF is a versatile cytokine with multifunctional roles in inflammation and cell death. TNFR1, as one of the receptors for TNF-α, is a death receptor and involved in the classical apoptosis (29). In order to investigate the inflammatory response to *M.tb* infection, serum levels of inflammatory factors were assessed by ELISA in patients with spinal tuberculosis, with healthy volunteers serving as the control group. The results revealed a significant upregulation of serum concentrations of IL-6 and TNF-α in patients with spinal tuberculosis compared to healthy controls (**Figure 2A**). Immunofluorescence staining of TUNEL and VDR was conducted, showing strong positive signals in patients with spinal tuberculosis compared to healthy controls (**Figure 2B**). These findings suggest that *M.tb* infection leads to increased expression of TNF-α, IL-6, and severe apoptosis.

### 2.3 1,25(OH)_2_D_3_ is in a dose-dependent manner inhibits *M.tb*-induced inflammation

**Figure 3:**
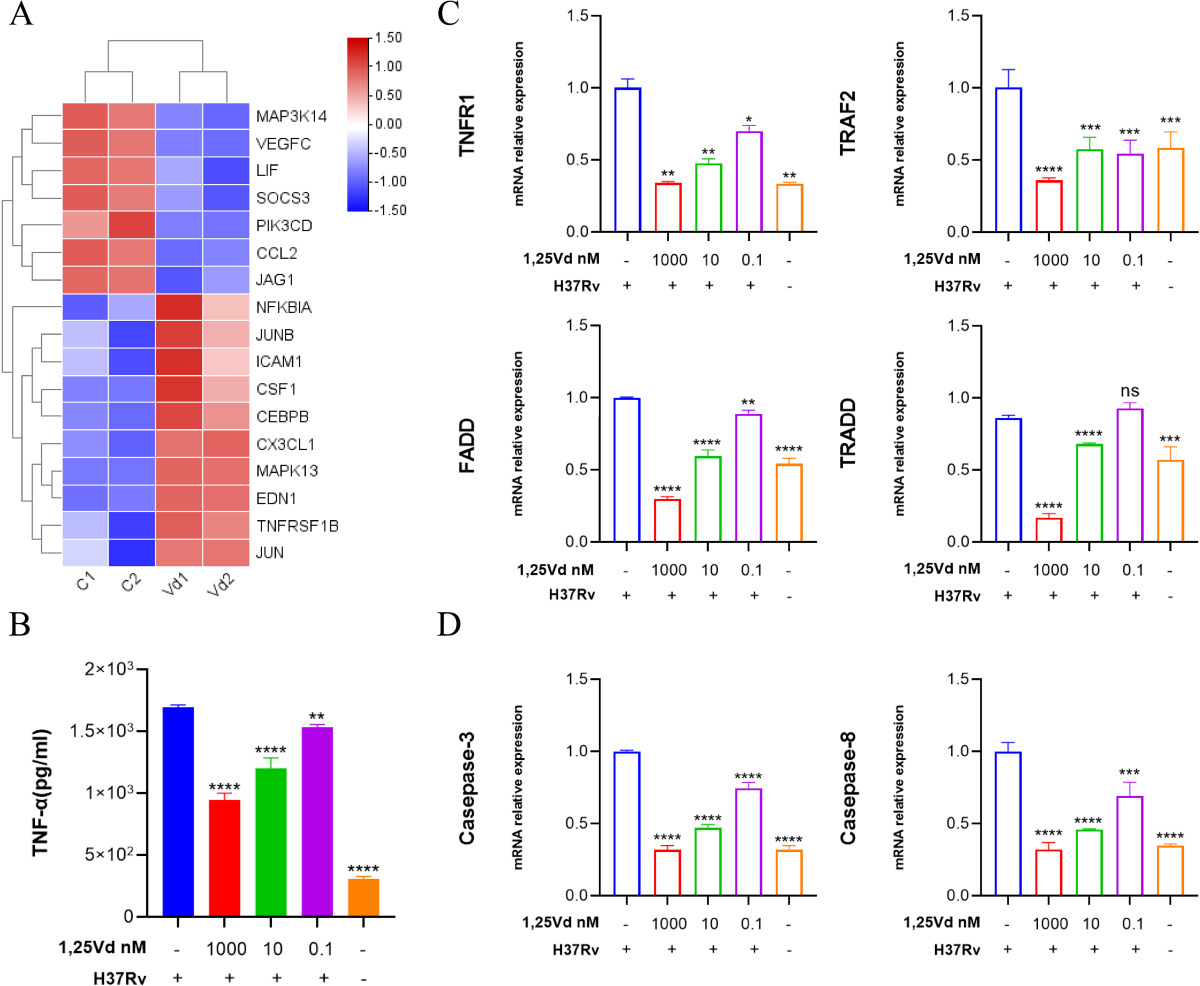
1,25(OH)_2_D_3_ dose-dependently inhibits *M.tb*-induced secretion of inflammatory factor. **(A)** The heat map shows both upregulated and downregulated genes in the TNF pathway with 1,25(OH)_2_D_3_ intervention in infected THP-1 cells. **(B)** The level of TNF-α in the supernatant was measured after treating the infected THP-1 cells with 1,25(OH)_2_D_3_ for 24 hours by Elisa. (n=3) **(C, D)** After 24 hours of 1,25(OH)_2_D_3_ treatment on infected THP-1 cells, the six genes transcriptional levels of TNF and apoptosis pathway were detected. (n=3) One-way ANOVA was used to calculate p values. *p < 0.05, **p < 0.01, ***p < 0.001, ****p<0.0001.

To identify the effector molecules regulated by 1,25(OH)_2_D_3_, *M.tb*-infected cells were treated with a gradient concentration of 1,25(OH)_2_D_3_ for 24 hours. The cell supernatant was collected to detect the levels of TNF-α and cells were collected for high-throughput sequencing. The differential genes related to the TNF signaling pathway were visualized using a heat map (**Figure 3A**). Compared with the untreated group, different concentrations of 1,25(OH)_2_D_3_ could significantly restrain the secretion of TNF-α dose-dependently (**Figure 3B**). These results indicate that 1,25(OH)_2_D_3_ can suppress the *M.tb*-induced inflammatory response in a concentration-dependent manner. Meanwhile, qRT-PCR was performed to validate the sequencing results and identify specific genes associated with the TNF signaling pathway and apoptosis, as well as to assess the regulatory effect of 1,25(OH)_2_D_3_ in infected cells. Results indicated that *M.tb* infection markedly increased the mRNA expression of TNFR1, TRAF2, FADD, and TRADD, activating the TNF signaling pathway. Conversely, 1,25(OH)_2_D_3_ suppressed the aforementioned genes dose-dependently (**Figure 3C**). Additionally, *M.tb* infection significantly upregulated the mRNA expression of Caspase-3 and Caspase-8, thereby inducing apoptosis in macrophages. In contrast, 1,25(OH)_2_D_3_ exhibited an inhibitory effect on *M.tb*-induced apoptosis (**Figure 3D**). The above results provide preliminary evidence that 1,25(OH)_2_D_3_ inhibits *Mtb*-induced inflammation and apoptosis through the TNF signaling pathway in THP-1 cells.

### 2.4 The effects of 1,25(OH)_2_D_3_ on the apoptosis of infected THP-1 cells

**Figure 4:**
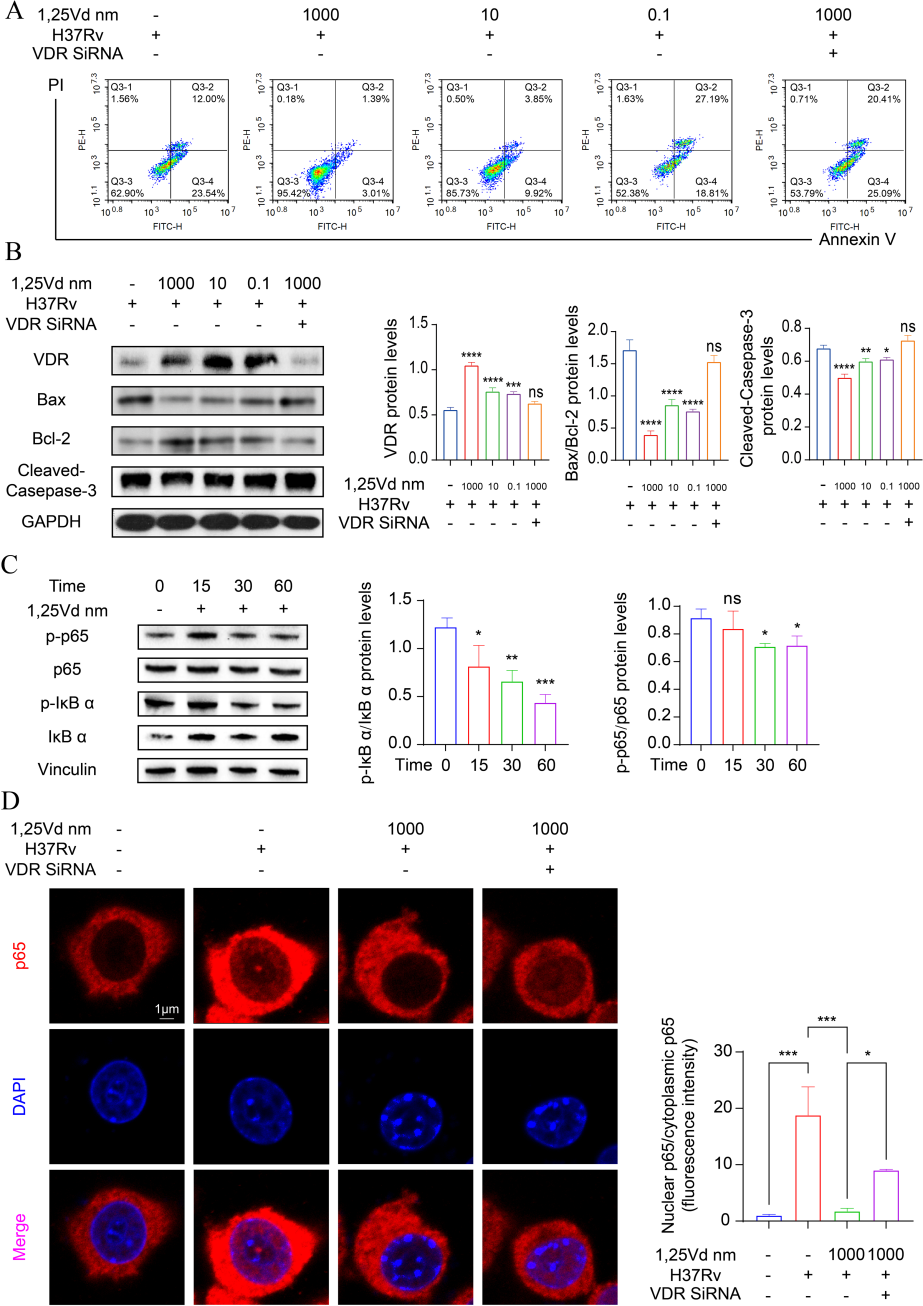
1,25(OH)_2_D_3_ inhibits *M.tb*-induced apoptosis by hindering p65 to entry into the nucleus. **(A)** The apoptosis level of infected THP-1 cells treated with 1,25(OH)_2_D_3_ for 24 hours was detected by flow cytometry. (n=3) **(B, C)** Protein levels of infected THP-1 cells treated with 1,25(OH)_2_D_3_ for 24 hours were analyzed by Western blot. Data are normalized to β-actin and presented as mean ± SD. (n=3) **(D)** Immunofluorescence staining images of p65 (red), DAPI (blue) and the merged. Scale bars, 1µm. (n=3) One-way ANOVA was used to calculate p values. *p < 0.05, **p < 0.01, ***p < 0.001, ****p<0.0001. ns, non-significant.

To elucidate the effects of 1,25(OH)_2_D_3_ on the apoptosis of infected THP-1 cells, flow cytometry was applied for apoptosis detection in THP-1 cells 24h after infection. First, we performed the CCK-8 assay to determine the cytotoxic effects of varying concentrations of VD on PMA-treated THP-1 cells within 24 hours (**Supplementary Figure S1**). The results revealed that Treatment with 1,25(OH)_2_D_3_ led to a significant inhibition of early apoptosis in infected cells compared to the control group (P<0.01). Specifically, 1,25(OH)_2_D_3_ appeared to reduce early apoptosis by approximately 3.01% at a concentration of 10^-6^ M (**Figure 4A**, **Supplementary Figure S2**). Western blot analysis indicated that 1,25(OH)_2_D_3_ notably increased VDR expression and suppressed apoptosis by downregulating Bax and upregulating Bcl2, ultimately inhibiting activated caspase-3. To explore the role of VDR, VDR siRNA was used to silence the expression of VDR in THP-1 cells, then pretreatment with 10^-6^ mol/L concentration of 1,25(OH)_2_D_3_ for 24 hours, followed by *M.tb* infection for 4 hours. Results showed the effect mediated by 1,25(OH)_2_D_3_ was reversed upon VDR silencing (**Figure 4B**). To further explore the underlying mechanism, THP-1 cells were exposed to *M.tb* for 4 hours, followed by treatment with 1,25(OH)_2_D_3_ (10^-6^ mol/L) for 0, 15, 30, and 60 minutes. Western blot results indicated that treatment with 1,25(OH)_2_D_3_ greatly reduced the ratio of p-IκB α/IκB α and p-p65/p65 (**Figure 4C**). Additionally, the immunofluorescence staining for p65 was carried out, demonstrating a marked increase in nuclear translocation of p65 upon *M.tb* treatment, whereas 1,25(OH)_2_D_3_ evidently suppressed its translocation. Notably, nuclear translocation of p65 was reinstated following VDR silencing (**Figure 4D**). These findings suggest that 1,25(OH)_2_D_3_ inhibited *M.tb*-induced apoptosis in THP-1 cells through the NF-κB signaling pathway.

### 2.5 1,25(OH)_2_D_3_ attenuated *M.tb*-induced cytokine storm-associated multiple organ damage *in vivo*

**Figure 5:**
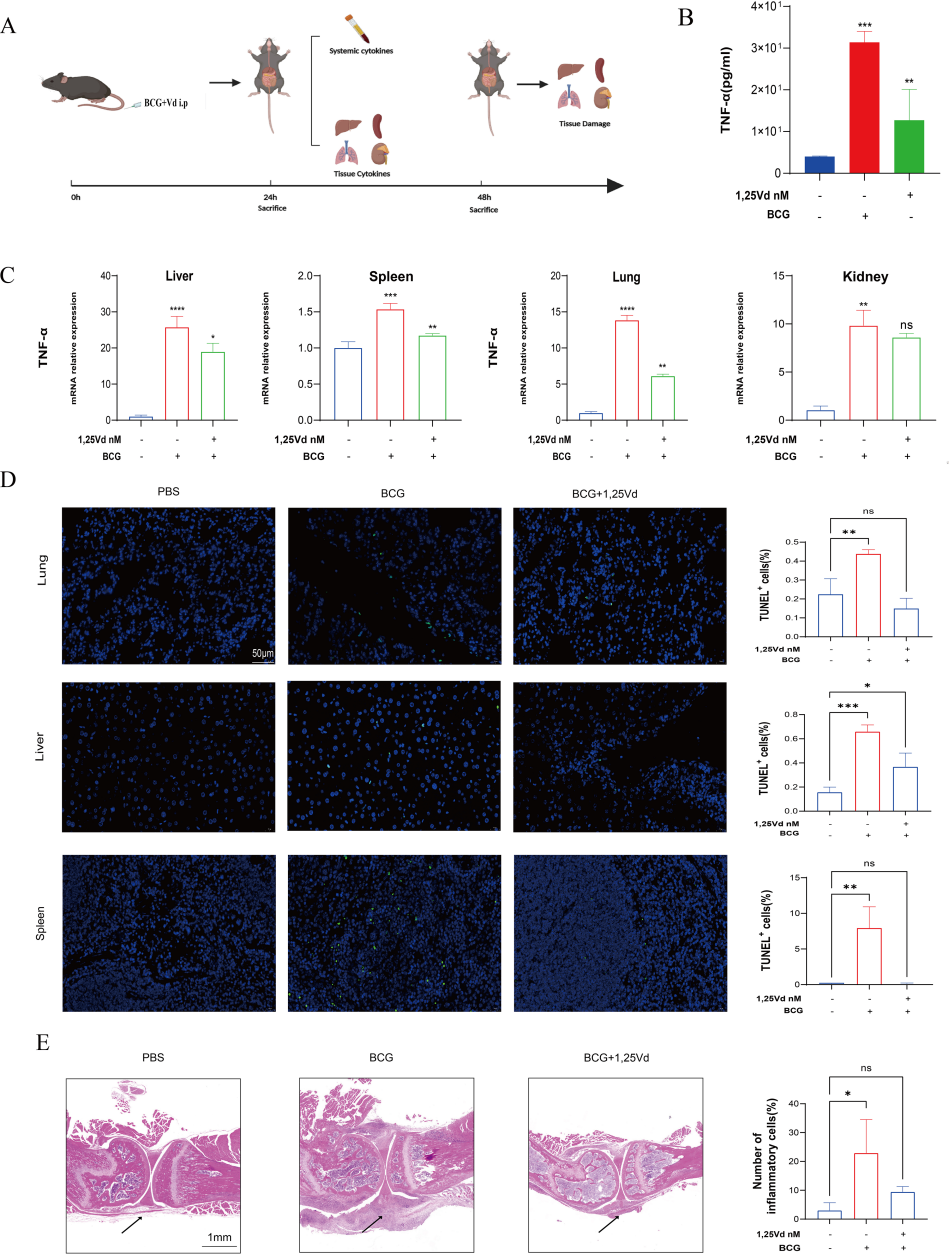
1,25(OH)_2_D_3_ inhibits BCG -induced inflammatory response *in vivo*. **(A)** Schematic illustration of animal experiments. **(B)** The level of TNF-α in the serum of mice was detected by ELISA after treatment with 1,25(OH)_2_D_3_ and BCG infection for 24 hours. (n=5) **(C)** The gene expression levels of TNF-α in the liver, spleen, lung, and kidney of mice were detected after treatment with 1,25(OH)_2_D_3_ and BCG infection for 48 hours. (n=5) **(D)** Representative TUNEL staining micrographs of the lung, liver, spleen and kidney sections at 48 h after BCG challenge (scale bar, 50μm). (n=5) **(E)** Representative *H&E* staining micrographs of knee joint sections at 48 h after BCG challenge (scale bar, 1mm). (n=3) One-way ANOVA was used to calculate p values. *p < 0.05, **p < 0.01, ***p < 0.001, ****p<0.0001.

*M.tb* infection of macrophages typically localizes to different parts of the body via the bloodstream, leading to the development of various extrapulmonary tuberculosis diseases. To validate the results, we further explored the effects of 1,25(OH)_2_D_3_ *in vivo* using a mouse model of *M.tb* infection through intravenous injection (**Figure 5A**) (30, 31). Due to the animal laboratory’s limited security level of P2+, we utilized BCG for the animal experiments. To mimic this process, we administered BCG and 1,25(OH)_2_D_3_ via tail vein injection. 24h post-injection, we obtained ocular blood samples from the mice to measure TNF-α levels using ELISA. The results demonstrated a substantial increase in TNF-α levels in the blood of mice following BCG infection (P<0.001). Additionally, treatment with 1,25(OH)_2_D_3_ markedly decreased the expression of TNF-α (P<0.01) (**Figure 5B**). In order to assess the expression of TNF-α in organs, tissue samples from the lungs, livers, spleens, and kidneys of all mouse groups were collected for RNA extraction and subsequent qRT-PCR analysis. The findings revealed a substantial and consistent elevation in TNF-α transcript levels in multiple organs following BCG infection across all groups of mice. Furthermore, treatment with 1,25(OH)_2_D_3_ led to a notable decrease in TNF-α transcript levels in the lungs, liver, and spleen, but not in the kidneys (**Figure 5C**). *H&E* staining results did not show a significant inflammatory response among these groups, potentially due to the short duration of the infection or the weak virulence of the BCG (**Supplementary Figure S1**). To assess the effect of 1,25(OH)_2_D_3_ on apoptosis *in vivo*, immunofluorescence staining for TUNEL was conducted on liver, spleen and lung sections from all groups of mice. The results indicated that treatment with 1,25(OH)_2_D_3_ markedly attenuated apoptosis induced by BCG infection in various tissues (**Figure 5D**). These findings indicate that 1,25(OH)_2_D_3_ inhibited *M.tb*-induced inflammation and apoptosis in various organs *in vivo*. We demonstrated *in vivo* and *in vitro* that 1,25(OH)_2_D_3_ ameliorates the inflammatory milieu induced by *M.tb* infection. Next we explored the effect of 1,25(OH)_2_D_3_ on *M.tb*-induced osteolysis via a mouse model of joint tuberculosis. BCG and 1,25(OH)_2_D_3_ were injected into the joint cavity of mice via a microsyringe. *H&E* staining results showed that treatment of BCG resulted in massive inflammatory cell infiltration and 1,25(OH)_2_D_3_ effectively inhibited BCG-induced inflammation (**Figure 5E**). These findings illustrate that 1,25(OH)_2_D_3_ effectively ameliorates *M.tb*-induced inflammation and multiple organ damage *in vivo*.

## 3 Discussion

*M.tb* is an intracellular bacterium that parasitizes macrophages and is mainly transmitted by aerosols. Therefore, the innate immunity of the lungs plays a crucial role in preventing the transmission of tuberculosis. Macrophages, as the first line of defense in innate immunity, are responsible to invasion by pathogenic organisms (32) and play a significant role in *M.tb* infection. We established a model of *M.tb* infection using THP-1 cells *in vitro* and conducted high-throughput sequencing, which revealed 10,000 differentially expressed genes, as illustrated in the volcano and heat maps. The KEGG pathway enrichment analysis highlighted the TNF signaling pathway as the most significantly affected. TNF-α, a versatile cytokine, is recognized for its pivotal role in immune inflammation and cell death (29). As TNF signaling pathway has been associated with proapoptotic signaling (33), the production of TNF-α induced by *M.tb* may be involved in triggering macrophage apoptosis. To investigate this further, we collected blood samples from patients with spinal tuberculosis and assessed the concentrations of IL-6 and TNF-α. The findings revealed a substantial increase in the inflammatory factors (TNF-α and IL-6) in the blood of infected individuals, indicating a robust inflammatory response associated with *M.tb* infection.

Inflammation is a crucial immune defense mechanism in the body, serving as a self-protective and tissue repair response to environmental stress or bacterial infection. While excessive inflammation can result in cellular damage (34). Immunofluorescence results of tissues from patients show that infection leads to elevated VDR expression and promotes apoptosis. These findings were further validated through *in vitro* experiments, where THP-1 cells were infected with *M.tb* strains and pre-treated with varying doses of 1,25(OH)_2_D_3_. High-throughput sequencing revealed the differential expression of 55 genes in the TNF signaling pathway. ELISA results showed a significant, dose-dependent reduction in TNF-α levels following pre-treatment with 1,25(OH)_2_D_3_. Consistently, qPCR results indicated a marked decrease in the expression of TNF-related genes upon *M.tb* infection of THP-1 cells, suggesting inhibition of the TNF signaling pathway. *M.tb* activates the NF-κB inflammatory signaling pathway by triggering Myd88 through TLR2. The NF-κB signaling cascade is essential to the inflammatory response in macrophages (35). Our results indicate that *M.tb* infection promotes the phosphorylation of NF-κB p65 and its translocation into the nucleus, and this process is inhibited by 1,25(OH)_2_D_3_. It’s reported that vitamin D can enhance macrophage-mediated killing of *M.tb* through the activation of both the VDR and antimicrobial peptide pathways (36). Consequently, the retention of *M.tb* within macrophages may prove to be a more efficacious strategy in promoting the clearance of pathogenic bacilli by the host. Following infection with *M.tb*, vitamin D provides protection by reducing the secretion of inflammatory factors, inhibiting apoptosis, and augmenting the bactericidal activity of macrophages.

Within the bone microenvironment, macrophages have the capacity to differentiate into osteoclasts and contribute to the bone remodeling process. Inflammation in the bone microenvironment can lead to the loss of bone mass by triggering excessive osteoclastogenesis (37). It’s reported that intracellular infection with *M.tb* can lead to the aberrant activation of osteoclasts, ultimately leading to substantial bone loss. Disrupted apoptosis of any cell within the bone microenvironment can result in an imbalance of bone homeostasis (38). Therefore, it is crucial to maintain the stability of bone homeostasis for the effective treatment of bone tuberculosis. *M.tb* employs distinct pro-apoptotic and anti-apoptotic mechanisms, both promoting and inhibiting apoptosis to facilitate intracellular survival in human alveolar macrophages (39). Apoptotic pathways are classified as intrinsic and extrinsic (40). The extrinsic pathway entails the activation of Caspase-8, ultimately triggering the activation of Caspase-3 to initiate cell death. *M.tb* infection markedly elevated the expression of Caspase-3 and Caspase-8, which were inhibited by 1,25(OH)_2_D_3_. Bcl-2 and Bax both belong to the Bcl-2 family; Bcl-2 inhibits cell apoptosis, whereas Bax promotes cell apoptosis (41). As the dosage of 1,25(OH)_2_D_3_ increased, the results showed elevated expression of Bcl-2, reduced expression of Bax, and decreased expression of Cleaved-Caspase-3. Overall, we have demonstrated the role of 1,25(OH)_2_D_3_ in inhibiting apoptosis in infected macrophages. Moreover, we simulated the process of nodules circulating in the bloodstream by injecting BCG through the tail vein. Our findings revealed a significant reduction in the inflammatory cytokine TNF-α in the 1,25(OH)_2_D_3_-treated group of mice. While our earlier work established VD’s direct suppression of M.tb-induced osteoclastogenesis, this study reveals that VD also disrupts osteolytic inflammation by rescuing apoptosis and TNF-α dysregulation, offering a broader mechanistic framework for its therapeutic potential (25).

## 4 Materials and methods

### 4.1 Bacterial strains

The *M. tuberculosis H37Rv* standard strain (abbreviated as *M.tb*) was provided by Chongqing Public Health Medical Center, while the BCG strain was purchased from Shanghai Jingnuo. All strains were resuscitated on solid Middle Brook 7H11 medium (Becton Dickinson, USA, 283810) supplemented with 10% oleic-albumin-dextrose-catalase (OADC, Becton Dickinson, USA, 212351) and 0.5% glycerol (Solarbio, CHN, G8190). Individual colonies of *M.tb* and BCG were selected and cultured in Middlebrook 7H9 medium (Becton Dickinson, USA, 271310) supplemented with 10% OADC, 0.5% glycerol, and 0.05% Tween 80 (Sigma Aldrich, USA, P4780).

### 4.2 Cell culture and bacterial infection

The THP-1 cell lines were purchased from ATCC and maintained in 1640 medium (Gibco, USA,12633020) supplemented with 10% fetal bovine serum (Gibco, USA, A5670701) and 1% Penicillin & Streptomycin solution (Solarbio,CHN,P1400) in a 5% CO_2_ atmosphere at 37°C. The cells were seeded in 24-well plates at a density of 1 × 10^5^ cells/well and incubated with phorbol myristate (PMA, Sigma Aldrich, USA) at a concentration of 100 ng/ml overnight to ensure proper cell adherence. 1,25(OH)_2_D_3_ (Sigma-Aldrich, USA, d1530) with purity >95% was stored at −20°C in the light-protected condition. Adherent THP-1 cells were infected with *M.tb* at an MOI of 1 for 4 hours, and then washed three times with PBS to remove bacteria. Experiments involving live *M.tb* were performed in a P2+ facility.

### 4.3 RNA sequencing

Total RNA was isolated using the RNeasy Mini Kit (Qiagen, GER,74106). Paired-end libraries were synthesized using the TruSeq™ RNA Sample Preparation Kit (Illumina, USA, RS-122-2001) following the TruSeq™ RNA Sample Preparation Guide. In brief, poly-A containing mRNA molecules were purified using poly-T oligo-attached magnetic beads. Subsequently, the mRNA was fragmented into small pieces using divalent cations at 94°C for 8 min. The cleaved RNA fragments were then used to generate first-strand cDNA with reverse transcriptase and random primers. This was followed by the synthesis of second-strand cDNA using DNA Polymerase I and RNase H. The resulting cDNA fragments underwent an end repair process, the addition of a single ‘A’ base, and ligation of the adapters. The products were then purified and enriched with PCR to create the final cDNA library. The purified libraries were quantified using a Qubit^®^ 2.0 Fluorometer (Life Technologies, USA) and validated with an Agilent 2100 Bioanalyzer (Agilent Technologies, USA) to confirm the insert size and calculate the molar concentration. Clusters were generated by cBot with the library diluted to 10 pM and then sequenced on the Illumina NovaSeq 6000 (Illumina, USA).

### 4.4 ELISA quantification

In order to analyze the secretion of inflammatory factors in serum and cell supernatant, ELISA kits for TNF-α (NeoBioscience, CHN, 3511-1H-6) and IL-6 (NeoBioscience, CHN, 3361-1A-6) were utilized. In brief, 100 μL of sample or standard was added to each well of a 96-well plate, and then incubated with anti-TNF-α or anti-IL-6 at 37°C for 90 min. Each well was incubated with streptavidin-HRP at 37°C for 30 min. After incubating with TMB and stop solution, the 96-well plate was measured at 450 nm using a microplate reader (Thermo Multiskan FC, GER). The absorbance was measured at 450 nm with correction for non-specific background at 570 nm in accordance with the manufacturer’s instructions.

### 4.5 RNA extraction and quantitative reverse transcription polymerase chain reaction

Total RNA of tissues or cells was extracted using an RNA simple Total *RNA* Kit (ES science, CHN) and reverse‐transcribed (RT) with ReverTra Ace qPCR RT Master Mix (Takara, JAN). Quantitative reverse transcription polymerase chain reaction (PCR) was carried out using SYBR qPCR Mix (Takara, JAN). The reaction volume of the RT mixture was 20 μl, while the reaction volume of the PCR mixture was 20 μl (cDNA 1‐8 μl, MIX 10 μl, primer F 1 μl, primer R 1 μl, with DEPC H2O added to 20 μl). The experiments were conducted following the provided protocols. The sequences of the primers used are listed in **Table 1**.

**Table 1:** Primer sequences of genes.

Gene	Primer sequence
M-TNF-α	F: GCCTCCCTCTCATCAGTTCTATG
	R: ACCTGGGAGTAGACAAGGTACAA
H-TNFR1	F: GTCAGGTGGAGATCTCTTCTTG
	R: GAAGCACTGGAAAAGGTTTTCA
H-FADD	F: GACCGAGCTCAAGTTCCTATG
	R: GACCGAGCTCAAGTTCCTATG
H-TRAF2	F: GAACACACCTGTCCCTCTTCTTT
	R: CAATCACGTGCTCCCGGTTATT
H-TRADD	F: CGCTGTTTGAGTTGCATCCTAG
	R: CCGAGCCGCACTTCAGATTT
H-Caspase-8	F: TCAACAAGAGCCTGCTGAAGATA
	R: GGAGAGTCCGAGATTGTCATTAC
H-Caspase-3	F: TGAGCCATGGTGAAGAAGGAATAA
	R: CCCGGGTAAGAATGTGCATAAAT
H-GAPDH	F: GGAGTCCACTGGCGTCTTCA
	R: GTCATGAGTCCTTCCACGATACC

### 4.6 Immunofluorescence staining

The pathological tissues from spinal tuberculosis intervertebral discs (study group) and the control tissues (negative controls from intervertebral disc herniation patients) were collected from patients undergoing curettage of spinal tuberculosis and non-tuberculosis patients with ethical approval(Approval ID:KY2024198) and according to the Army Medical University and Human Tissue Act ethical procedures. Then Immunofluorescence staining was performed for TUNEL (Beyotime, CHN, C1086) and VDR (CST, USA,12550S). The tissues were sliced into 2-mm sections and treated with 0.1% Triton X-100 for 5 min and then blocked with 10% goat normal serum for at least 30 min. Then dilute the primary antibodies with 1:200 in blocking buffer to incubate the sections for 1 h at 37°C. Goat Alexa Fluor 488 anti-Rabbit IgG (Bioss, CHN, bs-0295G-BF488), Alexa Fluor 647 Goat anti-Mouse IgG (Bioss, CHN, bs-0296G-BF647) (1:500), and DAPI (Solarbio, CHN, C0065) (1:1000) were then applied. The fluorescence signal was observed under a fluorescence microscope.

### 4.7 Flow cytometry

Apoptosis rate was detected using the Annexin V-FITC apoptosis detection kits (Biospes, CHN, BAD1001). Briefly, 1 × 10 ^5^/Test of infected THP-1 cells were resuspended in 100 μl binding buffer, then labeled with Annexin V-FITC (5 μl) and propidium iodide (PI) (5 μl) for 150min in the dark at room temperature. After 1 h, the green (Annexin V-FITC) and red (PI) fluorescence were examined by flow cytometry (NovoCyte, ACEA, USA). The examination wavelength was 488 nm, and the emission wavelength was 530 nm.

### 4.8 Western blot analysis

Briefly, equal amounts of total protein (30 µg) from different groups of samples were loaded for each lane and separated by 10% SDS-PAGE, followed by electroblotting by transferring protein to the PVDF membrane. The primary antibody target on Caspase-3, Caspase-8, VDR, Bax, Bcl-2 and GAPDH were used, and HRP-conjugate goat anti-rabbit IgG as a secondary antibody. The bands’ intensities were quantified using Image J after being conversed with the grayscale. Anti-GAPDH (AP0063) antibody was purchased from Biworld (Minnesota, USA), Anti-Caspase-3 (A19654) antibody was purchased from ABclonal (Wuhan, China), Anti-Caspase-8 (ab32125) antibody was purchased from Abcam (Cambridge, Britain), Anti-VDR (12550S) antibody was purchased from CST (Boston, USA), Anti-Bax (5023S) antibody was purchased from CST (Boston, USA), Anti-Bcl-2 (4223S) antibody was purchased from CST (Boston, USA). HRP-conjugated anti-rabbit secondary antibody (A0208) was purchased from Beyotime Biotechnology (Shanghai, China).

### 4.9 *In vivo* model of BCG infection and 1,25(OH)_2_D_3_ treatments

Male C57BL/6 mice (20–25 g) were purchased from the animal center of the Army Medical University. A total of 15 6-week-old C57/BL6 male mice were randomly distributed into three equal groups (n=5): vehicle (injection with PBS), BCG (injection with 1×10^7^ CFU/mouse) and BCG +1,25(OH)_2_D_3_ (injection with 1×10^7^ CFU/mouse BCG followed by 0.5 µg/g 1,25(OH)_2_D_3_). All compounds were administered via the tail vein and articular cavity. For cytokine level detection, serum and tissues were collected from mice 24h after 1,25(OH)_2_D_3_ treatment. To assess organ damage, tissues including lung, liver and spleen were collected 48h after 1,25(OH)_2_D_3_ treatment. Hematoxylin and eosin (*H&E*) staining was then performed according to the manufacturer’s instructions and the sections were observed using a light microscope.

### 4.10 Statistics and analysis

All experimental data were analyzed using GraphPad Prism (version 9.0) and were shown as mean ± standard error of the mean (SEM). An unpaired Student’s t-test for two groups or one-way analysis of variance for multiple groups was used to analyze differences. The grey value of the Western blot protein bands was normalized with GAPDH or β‐actin. All experiments were independently repeated three times. Statistical significance was set at P<0.05.

## 5 Conclusion

Our findings demonstrate that 1,25(OH)_2_D_3_ exerts dual immunomodulatory effects during *M.tb* infection. Attenuation of pro-inflammatory TNF-α release coupled with suppression of macrophage apoptosis, and disruption of NF-κB signaling through inhibition of p65 phosphorylation and nuclear translocation. These coordinated mechanisms suggest that vitamin D signaling serves as a critical checkpoint balancing inflammatory response and programmed cell death in infected macrophages. Given the established role of macrophage apoptosis in both bacterial clearance (via efferocytosis) and antigen presentation, our discovery of 1,25(OH)_2_D_3_-mediated pathway regulation provides:

- Therapeutic Implications: A druggable target for adjunctive therapies to modulate immunopathology in active TB.
- Vaccine Development: Mechanistic insights for optimizing vaccine vectors that harness apoptosis-dependent antigen cross-presentation.
- Host-Directed Strategy: Molecular rationale for precision modulation of NF-κB dynamics in TB immunotherapies.

## Data Availability

All sequencing data has been uploaded to the GEO repository: https://www.ncbi.nlm.nih.gov/geo/query/acc.cgi?acc=GSE267737.

## References

[1] Visca D Ong CWM Tiberi S Centis R D’Ambrosio L Chen B. Tuberculosis and COVID-19 interaction: A review of biological, clinical and public health effects. Pulmonology. (2021) 27:151–65. doi: 10.1016/j.pulmoe.2020.12.012 PMC7825946 33547029

[2] Lee HJ Kim NH Lee EH Yoon YS Jeong YJ Lee BC. Multicenter testing of a simple molecular diagnostic system for the diagnosis of mycobacterium tuberculosis. Biosensors. (2023) 13:259. doi: 10.3390/bios13020259 36832025 PMC9954000

[3] Kanga I Taylor JA Jacobs C Outerbridge G. Tuberculosis of the neuromusculoskeletal system: a review of two cases presenting as chiropractic patients. J Can Chiropractic Assoc. (2015) 59:13–23. PMC4319452 25729081

[4] Ware CF. The TNF superfamily. Cytokine Growth Factor Rev. (2003) 14:181–4. doi: 10.1016/s1359-6101(03)00032-7 12787557

[5] Jain AK Rajasekaran S Jaggi KR Myneedu VP. Tuberculosis of the spine. J Bone Joint Surgery Am. (2020) 102:617–28. doi: 10.2106/JBJS.19.00001 32028313

[6] Shi T Li T Li J Wang J Zhang Z. Genetic diversity of drug resistant Mycobacterium Tuberculosis in local area of Southwest China: a retrospective study. BMC Infect Dis. (2018) 18:565. doi: 10.1186/s12879-018-3503-0 30428837 PMC6234635

[7] Zhang R Pu J Zhou J Wang Q Zhang T Liu S. Factors predicting self-report adherence (SRA) behaviours among DS-TB patients under the “Integrated model”: a survey in Southwest China. BMC Infect Dis. (2022) 22:201. doi: 10.1186/s12879-022-07208-6 35232384 PMC8889779

[8] Buchrieser J Oliva-Martin MJ Moore MD Long JCD Cowley SA Perez-Simón JA. RIPK1 is a critical modulator of both tonic and TLR-responsive inflammatory and cell death pathways in human macrophage differentiation. Cell Death Dis. (2018) 9:973. doi: 10.1038/s41419-018-1053-4 30250197 PMC6155173

[9] Schwander S Dheda K. Human lung immunity against Mycobacterium tuberculosis: insights into pathogenesis and protection. Am J Respir Crit Care Med. (2011) 183:696–707. doi: 10.1164/rccm.201006-0963PP 21075901 PMC3081283

[10] Rohde K Yates RM Purdy GE Russell DG. Mycobacterium tuberculosis and the environment within the phagosome. Immunol Rev. (2007) 219:37–54. doi: 10.1111/j.1600-065X.2007.00547.x 17850480

[11] Innate immunity in tuberculosis: myths and truth - PubMed(2023). Available online at (Accessed November 30).

[12] Chan FK-M Luz NF Moriwaki K. Programmed necrosis in the cross talk of cell death and inflammation. Annu Rev Immunol. (2015) 33:79–106. doi: 10.1146/annurev-immunol-032414-112248 25493335 PMC4394030

[13] Chow A Lucas D Hidalgo Andrés Méndez-Ferrer Simón Hashimoto D Scheiermann C. Bone marrow CD169+ macrophages promote the retention of hematopoietic stem and progenitor cells in the mesenchymal stem cell niche. J Exp Med. (2011) 208:261–71. doi: 10.1084/jem.20101688 PMC3039855 21282381

[14] Kaplan G Post FA Moreira AL Wainwright H Kreiswirth BN Tanverdi M. Mycobacterium tuberculosis growth at the cavity surface: a microenvironment with failed immunity. Infection Immun. (2003) 71:7099–108. doi: 10.1128/IAI.71.12.7099-7108.2003 PMC308931 14638800

[15] Coleman MT Maiello P Tomko J Frye LJ Fillmore D Janssen C. Early Changes by (18)Fluorodeoxyglucose positron emission tomography coregistered with computed tomography predict outcome after Mycobacterium tuberculosis infection in cynomolgus macaques. Infection Immun. (2014) 82:2400–4. doi: 10.1128/IAI.01599-13 PMC4019174 24664509

[16] Chen M Gan H Remold HG. A mechanism of virulence: virulent Mycobacterium tuberculosis strain H37Rv, but not attenuated H37Ra, causes significant mitochondrial inner membrane disruption in macrophages leading to necrosis. J Immunol (Baltimore Md: 1950). (2006) 176:3707–16. doi: 10.4049/jimmunol.176.6.3707 16517739

[17] Brenner D Blaser H Mak TW. Regulation of tumour necrosis factor signalling: live or let die. Nat Rev Immunol. (2015) 15:362–74. doi: 10.1038/nri3834 26008591

[18] Pacifico L Osborn JF Bonci E Pierimarchi P Chiesa C. Association between vitamin D levels and nonalcoholic fatty liver disease: potential confounding variables. Mini Rev Medicinal Chem. (2019) 19:310–32. doi: 10.2174/1389557518666181025153712 30360708

[19] Liu S Liu Y Wan Bo Zhang H Wu S Zhu Z. Association between vitamin D status and non-alcoholic fatty liver disease: A population-based study. J Nutr Sci Vitaminology. (2019) 65:303–8. doi: 10.3177/jnsv.65.303 31474679

[20] Liu PT Stenger S Li H Wenzel L Tan BH Krutzik SR. Toll-like receptor triggering of a vitamin D-mediated human antimicrobial response. Sci (New York N.Y.). (2006) 311:1770–3. doi: 10.1126/science.1123933 16497887

[21] Arora P Goyal A Natarajan VT Rajakumara E Verma P Gupta R. Mechanistic and functional insights into fatty acid activation in Mycobacterium tuberculosis. Nat Chem Biol. (2009) 5:166–73. doi: 10.1038/nchembio.143 PMC2644305 19182784

[22] McCann LM Beto J. Roles of calcium-sensing receptor and vitamin d receptor in the pathophysiology of secondary hyperparathyroidism. J Renal Nutr. (2010) 20:141–50. doi: 10.1053/j.jrn.2010.01.004 20303786

[23] Zemel MB. Regulation of adiposity and obesity risk by dietary calcium: mechanisms and implications. J Am Coll Nutr. (2002) 21:146S–51S. doi: 10.1080/07315724.2002.10719212 11999543

[24] Duncan RE Ahmadian M Jaworski K Sarkadi-Nagy E Sul HS. Regulation of lipolysis in adipocytes. Annu Rev Nutr. (2007) 27:79–101. doi: 10.1146/annurev.nutr.27.061406.093734 17313320 PMC2885771

[25] Deng J Yang Y He J Xie Z Luo F Xu J. Vitamin D receptor activated by vitamin D administration alleviates Mycobacterium tuberculosis-induced bone destruction by inhibiting NFκB-mediated aberrant osteoclastogenesis. FASEB J. (2021) 35:e21543. doi: 10.1096/fj.202100135R 34046950 PMC12315972

[26] Posa F Di Benedetto A Cavalcanti-Adam EA Colaianni G Porro C Trotta T. Vitamin D promotes MSC osteogenic differentiation stimulating cell adhesion and αVβ3 expression. Stem Cells Int. (2018) 2018:6958713. doi: 10.1155/2018/6958713 29681950 PMC5851411

[27] Ma R Fang L Chen L Wang X Jiang J Gao L. Ferroptotic stress promotes macrophages against intracellular bacteria. Theranostics. (2022) 12:2266–89. doi: 10.7150/thno.66663 PMC8899587 35265210

[28] Rodríguez-Hernández E Quintas-Granados LI Flores-Villalva S Cantó-Alarcón JG Milián-Suazo F. Application of antigenic biomarkers for Mycobacterium tuberculosis. J Zhejiang University-SCIENCE B. (2020) 21:856–70. doi: 10.1631/jzus.B2000325 PMC7670104 33150770

[29] Aggarwal BB Gupta SC Kim JiH. Historical perspectives on tumor necrosis factor and its superfamily: 25 years later, a golden journey. Blood. (2012) 119:651–65. doi: 10.1182/blood-2011-04-325225 PMC3265196 22053109

[30] Lin Da Xu W Hong P Wu C Zhang Z Zhang S. Decoding the spatial chromatin organization and dynamic epigenetic landscapes of macrophage cells during differentiation and immune activation. Nat Commun. (2022) 13:5857. doi: 10.1038/s41467-022-33558-5 36195603 PMC9532393

[31] Michaud Josée Naud J Ouimet D Demers C Petit J-L Leblond FA. Reduced hepatic synthesis of calcidiol in uremia. J Am Soc Nephrology : JASN. (2010) 21:1488–97. doi: 10.1681/ASN.2009080815 PMC3013518 20595682

[32] Liu Na Dong Z Zhu X Xu H Zhao Z. Characterization and protective effect of Polygonatum sibiricum polysaccharide against cyclophosphamide-induced immunosuppression in Balb/c mice. Int J Biol Macromolecules. (2018) 107:796–802. doi: 10.1016/j.ijbiomac.2017.09.051 28939510

[33] Xaus J Comalada M Valledor AF Lloberas J López-Soriano F Argilés JM. LPS induces apoptosis in macrophages mostly through the autocrine production of TNF-alpha. Blood. (2000) 95:3823–31. doi: 10.1182/blood.V95.12.3823.012k07_3823_3831 10845916

[34] Ren Y Liu Y Liu K Huo X Liu C Zhang Y. Discovery of therapeutic candidates for diabetic retinopathy based on molecular switch analysis: application of a systematic process. Oxid Med Cell Longevity. (2022) 2022(1):3412032. doi: 10.1155/2022/3412032 PMC8758313 35035658

[35] Ozinsky A Underhill DM Fontenot JD Hajjar AM Smith KD Wilson CB. The repertoire for pattern recognition of pathogens by the innate immune system is defined by cooperation between toll-like receptors. Proc Natl Acad Sci U S A. (2000) 97:13766–71. doi: 10.1073/pnas.250476497 PMC17650 11095740

[36] Gombart AF. The vitamin D–antimicrobial peptide pathway and its role in protection against infection. Future Microbiol. (2009) 4:1151. doi: 10.2217/fmb.09.87 19895218 PMC2821804

[37] Du Mi Pan W Duan X Yang P Ge S. Lower dosage of aspirin promotes cell growth and osteogenic differentiation in murine bone marrow stromal cells. J Dental Sci. (2016) 11:315–22. doi: 10.1016/j.jds.2016.03.009 PMC6395233 30894990

[38] Qiu M Tu L Zhao M Yang M Qi J Xie Ya. Ataxia-televangelist mutated (ATM)/ATR serine/threonine kinase (ATR)-mediated RAD51 recombinase (RAD51) promotes osteogenic differentiation and inhibits osteoclastogenesis in osteoporosis. Bioengineered. (2022) 13:4201–11. doi: 10.1080/21655979.2022.2026729 PMC8974111 35176943

[39] Briken V Miller JL. Living on the edge: Inhibition of Host Cell Apoptosis by Mycobacterium tuberculosis. Future Microbiol. (2008) 3:415–22. doi: 10.2217/17460913.3.4.415 PMC2650273 18651813

[40] Huang B Lin M Lu L Chen W Tan J Zhao J. Identification of mini-chromosome maintenance 8 as a potential prognostic marker and its effects on proliferation and apoptosis in gastric cancer. J Cell Mol Med. (2020) 24:14415–25. doi: 10.1111/jcmm.16062 PMC7753872 33155430

[41] Zhang Q-F. Ulinastatin inhibits renal tubular epithelial apoptosis and interstitial fibrosis in rats with unilateral ureteral obstruction. Mol Med Rep. (2017) 16:8916–22. doi: 10.3892/mmr.2017.7692 PMC5779974 28990075

