# Supplementary material for: Vitamin D inhibits apoptosis in THP-1 cells infected with mycobacterium tuberculosis through TNF signaling pathway
Source: Front Immunol. 2025 May 6;16:1525922. doi: 10.3389/fimmu.2025.1525922 (PMC12089043; doi:10.3389/fimmu.2025.1525922)
Supplement: Supplementary file 1 [file DataSheet1.docx]

Vitamin D inhibits apoptosis in THP-1 cells infected with mycobacterium tuberculosis through TNF signaling pathway apoptosis

Yusheng Yang, Jiezhong Deng, Pan Liu, Jinyue He, Jiulin Tan, Bo Yu, Yun Bai, Fei Luo, Jianzhong Xu, Zehua Zhang.

Zehua Zhang, Department of Orthopedic, Southwest Hospital, Army Medical University, [zhangzehuatmmu@163.com](mailto:zhangzehuatmmu@163.com)

**Fig.S1** Cytotoxicity of different concentrations 1,25(OH)_2_D_3_ in THP-1cells. (n=3)

One-way ANOVA was used to calculate p values in this figure. ns representative.

**Fig.S2** The effect of different concentrations 1,25(OH)_2_D_3_ on apoptosis rate of THP-1 cells infected with H37Rv (n=3).

One-way ANOVA was used to calculate p values in this figure. **p < 0.01, ****p＜0.0001.


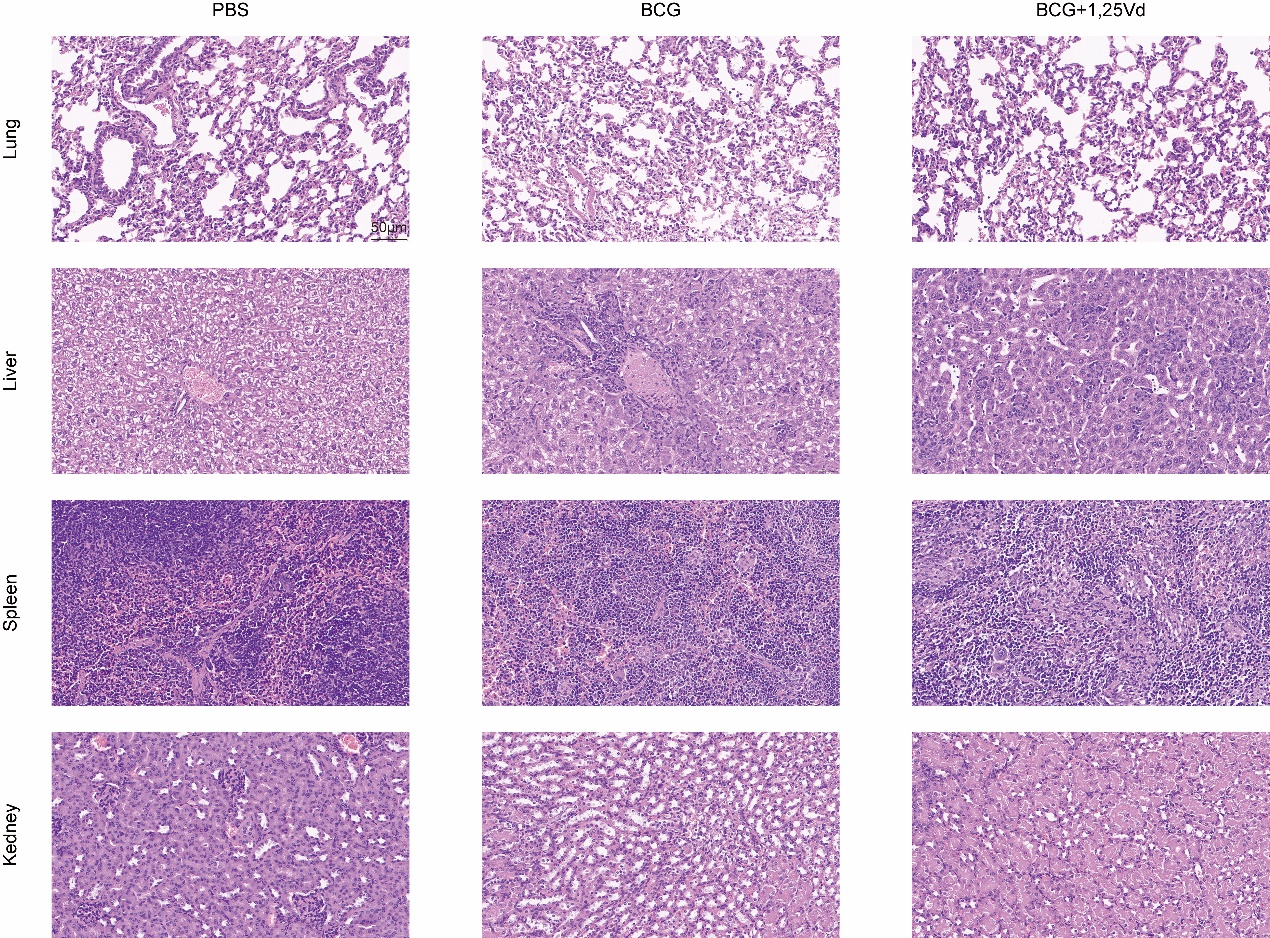


**Fig.S3** Histological evaluation of BCG on multiple organ damage in vivo. Hematoxylin and eosin (*H&E*) staining of liver, spleen, lung and kidney from mice of PBS, BCG and BCG+1,25(OH)_2_D_3_. Scale bar, 50 µm.(n=5)

Table 1. Primer sequences of genes.

| Gene | primer sequence |
| --- | --- |
| M-TNF-α | F: GCCTCCCTCTCATCAGTTCTATG |
|  | R: ACCTGGGAGTAGACAAGGTACAA |
| H-TNFR1 | F: GTCAGGTGGAGATCTCTTCTTG |
|  | R: GAAGCACTGGAAAAGGTTTTCA |
| H-FADD | F: GACCGAGCTCAAGTTCCTATG |
|  | R: GACCGAGCTCAAGTTCCTATG |
| H-TRAF2 | F: GAACACACCTGTCCCTCTTCTTT |
|  | R: CAATCACGTGCTCCCGGTTATT |
| H-TRADD | F: CGCTGTTTGAGTTGCATCCTAG |
|  | R: CCGAGCCGCACTTCAGATTT |
| H-Caspase-8 | F: TCAACAAGAGCCTGCTGAAGATA |
|  | R: GGAGAGTCCGAGATTGTCATTAC |
| H-Caspase-3 | F: TGAGCCATGGTGAAGAAGGAATAA |
|  | R: CCCGGGTAAGAATGTGCATAAAT |
| H-GAPDH | F: GGAGTCCACTGGCGTCTTCA |
|  | R: GTCATGAGTCCTTCCACGATACC |
